# Remission in moderate-to-severe pediatric asthma: Definitions, assessment, prevalence, predictors, and outcomes—a scoping review

**DOI:** 10.1016/j.jacig.2026.100786

**Published:** 2026-08-25

**Authors:** Cloé Laurier, Rébecca Fénélon-Dimanche, Sze Man Tse, Cristina Longo

**Affiliations:** a Centre de recherche Azrieli, Centre Hospitalier Universitaire Sainte-Justine, Montréal, Quebec, Canada; b Faculty of Medicine, Université de Montréal, Montréal, Quebec, Canada; c Faculty of Pharmacy, Université de Montréal, Montréal, Quebec, Canada

**Keywords:** Pediatric asthma, clinical remission, moderate-to-severe asthma, biologic therapies, scoping review, treat-to-target, disease control, airway inflammation, predictors, long-term outcomes

## Abstract

**Background:**

Remission is emerging as a novel therapeutic target in asthma, particularly with the advent of biologic therapies. Although spontaneous remission is recognized in mild childhood asthma, little is known about the concept of remission in children with moderate-to-severe asthma.

**Objective:**

This study aimed to synthesize available evidence on definitions, assessment methods, prevalence, predictors, and long-term outcomes of remission in children with moderate-to-severe asthma.

**Methods:**

This scoping review followed Preferred Reporting Items for Systematic Reviews and Meta-Analyses Extension for Scoping Reviews guidance. MEDLINE and EMBASE were searched from 2003 onward. Eligible studies included randomized clinical trials and observational studies involving children or adolescents with moderate-to-severe asthma that reported remission or remission-related subdomains.

**Results:**

Of 2076 records identified, 8 studies met inclusion criteria (1 trial, 7 observational). Definitions of remission varied substantially and predominantly relied on clinical criteria (absence of symptoms or medication use), with inconsistent incorporation of lung function and none including inflammatory biomarkers. Remission prevalence ranged widely, from 4% to 41.9%, with the highest rates observed in a recent biologic trial; however, assessment periods were highly variable, ranging from 24 weeks to 3 years. Predictors of a lower likelihood of remission included female sex, atopy, and older age at initial diagnosis. Available evidence suggests a persistent risk of subclinical inflammation and relapse despite achieving clinical remission.

**Conclusions:**

Evidence on remission in moderate-to-severe pediatric asthma remains limited and heterogeneous, often in the context of ongoing treatment. Standardized, multidomain definitions are urgently needed to inform treat-to-target strategies and future clinical trials.

Asthma is the most common chronic respiratory disease in children, affecting approximately 9.1% of children and 11.0% of adolescents globally.^1^^,^^2^ Within this population, moderate asthma is generally defined as disease requiring medium-dose inhaled corticosteroids (ICS) or low-dose ICS plus a second controller therapy such as long-acting *β*-2 agonists to maintain control, whereas severe asthma (affecting 5% to 10% of patients) remains uncontrolled or requires high-dose ICS and an additional controller therapy.^1^^,^^3^ Children with moderate-to-severe asthma face a considerable disease burden, including reduced quality of life, missed school days, frequent emergency visits and life-threatening exacerbations.^4^ Recurrent exacerbations, especially in those aged 6-11 years old, are associated with progressive lung function decline,^5^ increasing the risk of chronic obstructive pulmonary disease in adulthood.^6^^,^^7^

As evidence of the negative long-term effects of poor control in early life accumulates, asthma management remains largely reactive, with treatment adjustments made in response to symptom worsening.^8^ This exposes children to prolonged uncontrolled symptoms and exacerbations, increasing the risk of irreversible airway damage by delaying optimal control.^9^ In contrast, treat-to-target strategies, such as those implemented in autoimmune diseases, involve continuous monitoring and adjusting therapies until a predefined clinical goal is reached.^10^ This proactive framework promotes aggressive management to achieve outcomes like remission.

Although asthma remission is gaining attention as a treatment goal, particularly with the emergence of newer biologics, defining remission in children remains challenging.^11^ Remission is well described in mild asthma, where it often occurs spontaneously, as shown in long-term cohorts such as the Tucson Children’s Respiratory Study and Childhood Asthma Management Program (CAMP).^12^^,^^13^ However, little is known about the likelihood or predictors of remission in children with moderate-to-severe asthma, which is more persistent and treatment dependent.^14^

Remission categories have been proposed.^1^^,^^15^, ^16^, ^17^ Clinical remission is characterized by cessation of symptoms and exacerbations regardless of treatment. A more stringent threshold is complete remission, which requires normal pulmonary function without pharmacologic intervention, whereas biological remission is defined by low levels of airway inflammation and normalized airway responsiveness^14^^,^^15^ However, these definitions are derived from adult populations. Pediatric-specific factors such as lung development, treatment response, and disease heterogeneity are often overlooked.^18^ On-treatment remission, defined as the absence of symptoms and exacerbations while on therapy, may have distinct predictors and long-term implications compared with spontaneous, off-treatment remission in children with moderate-to-severe asthma.

A recent scoping review on pediatric asthma remission did not distinguish disease severity.^19^ Our work addresses this gap by focusing on moderate-to-severe asthma, a subgroup with more persistent disease, higher treatment burden, and increasing biologic use. In this population, remission is less likely to be spontaneous and more likely treatment associated, with distinct mechanisms and outcomes.

This scoping review examines evidence on both on-treatment and off-treatment remission in children with moderate-to-severe asthma, including definitions, assessments, prevalence, and associated factors. Specific questions include (1) What are the definitions and assessment methods of on-treatment and off-treatment remission across developmental stages, severity, and sex? (2) What are the predictors and long-term outcomes across these factors?

## Methods

This scoping review followed a framework guided by the Preferred Reporting Items for Systematic Reviews and Meta-Analyses Extension for Scoping Reviews statement and checklist, building on Preferred Reporting Items for Systematic Reviews and Meta-Analyses guidelines.^20^

### Search strategy and eligibility criteria

The search strategy was developed with a librarian from Université de Montréal (TS Hoang). Key target articles were compiled to ensure capture of relevant studies. MEDLINE, EMBASE, and the Cochrane Central Register of Controlled Trials were searched using Medical Subject Heading terms and keywords covering asthma, severity, treatment, and remission. Remission-related terms included recovery of function and absence of symptoms. Search results were managed in Covidence.

Eligible designs included trials and observational studies. Exclusion criteria were: articles not published in English or French, animal studies, case reports, case series, editorials, narrative reviews, or studies limited to mild asthma. Studies including both adults and children were eligible only if pediatric data were reported separately. The search was restricted to studies published from 2003 onward, when major clinical guidelines began standardizing asthma diagnosis and management across age groups. Duplicates were removed.

Two reviewers (C.L. and R.F.-D.) independently screened titles, abstracts, and full texts. A pilot screening ensured consistency. Discrepancies were resolved by discussion or a third reviewer (C.L.).

### Data charting and synthesis

The same reviewers independently extracted study characteristics and population, definitions of severity and remission, treatments, outcomes, prevalence statistics, predictors (if available), and limitations using a standardized form. Discrepancies were resolved by third reviewer. Remission was classified as a composite outcome across 5 subdomains: symptom control, exacerbations, medication use, lung function, and eosinophilic inflammation.^16^ Individual remission subdomains were also analyzed separately.

Subgroup analyses according to asthma severity, age groups (preschoolers, school-age, and adolescents), and sex were planned if sufficient data were available. Because asthma severity groupings (moderate vs severe) were not consistently defined across studies, proxy measures were used if available. In studies in which severity was not explicitly reported, poor asthma control was used as a proxy for greater disease severity. For example, in the study by Longo et al,^21^ remission prevalence was recalculated by combining estimates from published asthma control trajectory proportions. Children belonging to the out of control throughout, worsening control, and fluctuating control trajectories were classified as having suboptimal control, reflecting greater severity, whereas those in the consistently controlled or improving control trajectories were classified as having optimal control and milder severity. This categorization was undertaken to align with the protocol-specified subgroup analysis by disease severity, with suboptimal asthma control serving as a proxy for more severe disease.

For the synthesis of evidence on remission predictors, studies reporting effect estimates from multiple independent datasets had their dataset-specific estimates combined using random-effects meta-analysis to provide a single summary estimate and facilitate interpretation.

## Results

A total of 2076 studies were identified (Fig 1). After screening 1407 titles and abstracts, 84 full-text articles were assessed, of which 74 were excluded, leaving 8 studies to be synthesized.

**Fig 1:**
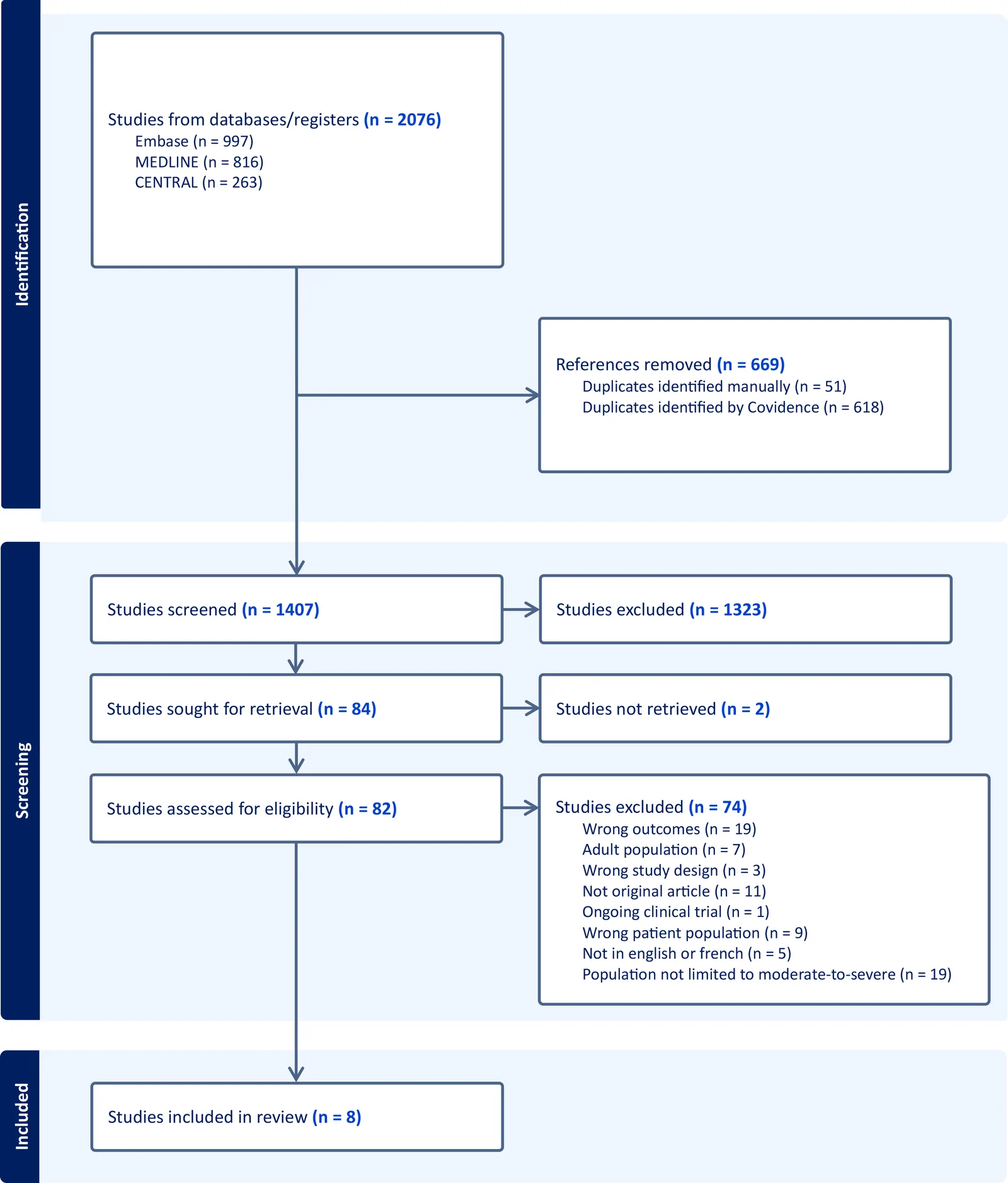
Flow chart of studies included in the scoping review.

### Study characteristics

Of the 8 studies meeting the inclusion criteria (Table I),^21^, ^22^, ^23^, ^24^, ^25^, ^26^, ^27^, ^28^ 1 was a randomized controlled trial, 1 was an open-label randomized controlled trial extension, and 6 were observational studies (cross-sectional [n = 3], prospective cohort [n = 2], and retrospective cohort [n = 1]). Most were published in the past decade (2006-2024) across North America (n = 4), Asia (n = 2), and Europe (n = 2).

**Table I:** Study characteristics of included studies investigating remission in pediatric moderate-to-severe asthma

Authors	Study design	Country	Study population (n)	Remission defined as a primary or secondary outcome	Population demographics	Baseline asthma medication use (ICS, rescue therapy, OCS)	Biologic therapy administered	Symptoms questionnaire score	Exacerbations (previous 12 mo) or rate	Lung function (%)	Total duration (mo)
Pumputiene et al (2006)^24^	Cross-sectional	Lithuania	83	Secondary	Mean age 8.73; 47% male; among the full sample, 35% had moderate persistent asthma (GINA guidelines)	History of ICS or leukotriene modifiers but ICS stopped ≥2 wk before study, no OCS ≥ 2 mo before study	None	NA	NA	NA	NA
Manuyakorn et al (2008)^25^	Cross-sectional	Thailand	65	Secondary	Mean age 12.7; 55% male asthma group and 50% male control group; 63% of children had moderate asthma out of all children with persistent asthma (GINA guidelines)	History of ICS treatment (mean duration for moderate asthma 6.6 y and asthma in remission 3.2 y)	None	NA	NA	FEV_1_ predicted: 83.63%	NA
Covar et al (2011)^26^	Prospective cohort	USA	909	Primary	Mean age 8.9; 61% male; 52.1% of children had moderate persistent asthma (clinical and spirometry assessment per CAMP criteria)	Approximate mean a of 0.67 annual prednisone course rate	None	NA	Approximate mean of 0.011 annual urgent care visit rate	Pre-BD FEV_1_= 94.5; post-BD FEV_1_ = 102.7; FVC = 103.6	67
Andersson et al (2013)^27^	Prospective population-based cohort	Sweden	248	Primary	Basely age 7-8 y; 19 y at end point (no mean age); 58% male; 35% of children had moderate asthma (asthma severity score of ≥2 of 5, based on symptoms and medication)	ICS use = 49% of children	None	NS	NA	Pre-BD FEV_1_ = 88	132-144
Odajima et al (2017)^23^	Prospective cohort	Japan	38	Secondary	Mean age 11.1; 60.5% male; 100% of children had severe uncontrolled allergic asthma (JPGL, 2008)	Mean ICS dose = 459.2 μg/d; 36% used asthma long-term control medications; 0% OCS	15% every 2 weeks and 23% every 4 wk had a dosing regimen of Omalizumab	JPAC	1.4	Pre-BD FEV_1_ = 90	10
DeBoer et al (2018)^28^	Cross-sectional	USA	187	Secondary	Mean age 11.3 male, 11.8 female; 62% male; high proportion African; American (∼41%); 59% had severe asthma (ERS/ATS criteria)	High-dose ICS ∼71%, low- to medium-dose ICS = 20%; 8%-5% Systemic OCS (current and past 3 mo)	None	ACQ-6 score	NA	Pre-BD FEV_1_ = 91.7; post-BD FEV_1_ = 106.5; FVC = 103.9	NA
Longo et al (2021)^21^	Retrospective population-based cohort	Canada (BC, SK, MB, QC)	48 687	Primary	69.2% Aged <3 y at diagnosis; 65.4% male; 42.5% had moderate to severe	ICS with or without other controllers = 68.3%; other non-ICS controllers = 4.6%	None	NA	4.7% to 4.2%		72
Bacharier et al (2024)^22^	RCT	Multinational (includes Canada, USA, Europe)	350	Primary	Age 6-11 y (no mean age); 64.1%-68.4% male; all children had moderate-to-severe asthma (GINA guidelines)	NA	Dupilumab	ACQ-5 score	2.2-2.6 (±1.6-2.9)	Pre-BD FEV_1_ = 76.4-78.4	12

General characteristics, baseline demographics, and definitions of asthma severity across the 8 included studies.

*ACQ5*, Asthma control questionnaire version 5; *ATS,* American Thoracic Society; *BC*, British Colombia; *ERS*, European Respiratory Society; *GINA*, Global INitiative for Asthma; *ICS*, inhaled corticosteroids; *JPGL*, Japanese Pediatric Guidelines for the Treatment and Management of Asthma; *MB*, Manitoba; *SK*, Saskatchewan; *NA*, not applicable/the variable was not measured or reported in the stud; *pre-BD*, prebronchodilator; *post-BD*, postbronchodilator; *QC*, Quebec; *OCS*, oral corticosteroids; *RCT*, randomized controlled trial.

Sample sizes varied from 38 to >900 participants, except 1 population-based cohort study utilizing administrative health data (∼48,000 children). Most included children aged 6-18 years (mean age 8-13), with a slight male predominance.

Definitions of asthma severity and baseline control varied substantially: studies rarely used a uniform “moderate-to-severe” classification and studies combined moderate and severe asthma or evaluated them separately. Baseline disease control, critical for predicting or comparing remission outcomes, was explicitly restricted to uncontrolled disease in some cohorts but was mixed or undefined in others.

Three studies (37.5%) evaluated combined moderate-to-severe asthma populations. Bacharier et al^22^ examined explicitly uncontrolled moderate-to-severe asthma using stringent criteria: requiring ≥3 months of stable medium-to-high–dose ICS plus a second controller, a prebronchodilator FEV_1_ ≤ 95% predicted, and ≥1 severe exacerbation requiring systemic glucocorticoids or hospitalization within the previous year. Longo et al^21^ used administrative data to identify cohorts based on controller medication dispensing. Andersson et al^27^ employed an arbitrary 5-point severity score incorporating wheeze frequency, daily medication use, and sleep disturbances, interpreting a score of ≥2 as moderate to severe.

Two studies (25%) focused on severe asthma. Odajima et al^23^ targeted severe uncontrolled allergic asthma defined by the Japanese Pediatric Guidelines for the Treatment and Management of Asthma, requiring a 6-month history of ICS (200-800 μg/day fluticasone propionate equivalent) combined with at least 2 additional controller therapies (such as leukotriene receptor antagonists or long-acting *β*-2 agonists), despite remaining uncontrolled.^29^ DeBoer et al^28^ applied American Thoracic Society/European Respiratory Society criteria to define severe asthma based on high-dose ICS requirements, although baseline control was not restricted to uncontrolled disease.

Finally, 3 studies (37.5%) isolated moderate asthma subgroups. Pumputiene et al^24^ and Manuyakorn et al^25^ defined moderate persistent asthma based on Global Initiative for Asthma criteria. Another study applied the CAMP definition,^30^ identifying moderate persistent asthma at screening based on a clinical assessment of symptoms or bronchodilator use occurring more than twice weekly.^26^

The included studies were conducted across tertiary hospitals, specialty clinics, and multicenter networks. The most frequently reported comorbidities were allergic rhinitis, eczema, and obesity.

### Definitions and subdomains of remission

Definitions of remission varied considerably, ranging from strict, multiyear criteria to less rigorous or undefined timeframes (Table II).^21^, ^22^, ^23^, ^24^, ^25^, ^26^, ^27^, ^28^ Bacharier et al^22^ applied the most stringent composite definition over 12 months, requiring absence of symptoms (Asthma Control Questionnaire, ACQ-5 < 0.75), no oral corticosteroid (OCS) use, and normalization of lung function (z score > –1.64 for both FEV_1_ and FEV_1_/forced vital capacity) despite ongoing ICS/long-acting *β*-2 agonists. This aligned with complete remission as defined by Canonica et al.^17^ Andersson et al^27^ applied a rigorous off-treatment definition, requiring absence of wheeze and no use of asthma medication (no OCS, no ICS, or rescue medication) for 3 consecutive years. Manuyakorn et al^25^ and Pumputiene et al^24^ also used a multidomain definition over 12 months, requiring no symptoms and FEV_1_ ≥ 80% predicted, with Manuyakorn et al^25^ additionally requiring no ICS use. Over a 1-year period, Covar et al^26^ (CAMP cohort) defined remission as the absence of asthma, meaning no unscheduled medical contact for asthma-related events, school absences symptoms, or asthma medications. Relying on administrative data rather than specific questionnaires, Longo et al^21^ defined remission as the absence of asthma-coded hospitalizations or physician visits and no dispensed asthma medications for 2 years. Finally, Odajima et al^23^ used the Japanese Pediatric Asthma Control questionnaire, evaluating daytime and nighttime symptoms, activity limitations, rescue medication use, and recent exacerbation, where a score of 15 of 15 indicated complete control without specifying duration.^31^ DeBoer et al^28^ examined the lung function and symptom domains at one time point but did not explicitly define remission.

**Table II:** Subdomains included in asthma remission definitions in studies explicitly assessing remission

Study	Time	Number of subdomains	No medications			No exacerbation	Absence of symptoms	Normal lung function	Other	% Achieving remission
			No OCS	No ICS	No rescue medication					
Pumputiene et al (2006)^24^	12 mo	2					×	× ∗FEV_1_ ≥80%		NR
Manuyakorn et al (2008)^25^	12 mo	3		×			×	× FEV_1_ > 80% and FEV_1_/FVC > 80%		NR
Covar et al (2011)^26^	12 mo	3	×	×	×	×	×			4
Andersson et al (2013)^30^	36 mo	2	×	×	×		× Absence of wheezing			10
Odajima et al (2017)^23^	NR	1					× JPAC = 15/15			40
Longo et al (2021)^21^	24 mo	3	×	×	×	×			× Absence of hospitalization or physician visit for asthma	41.5
Bacharier et al (2024)^22^	12m	4	×				× ACQ5 < 0.75	× z Score > -1.64 for FEV_1_ and FEV_1_/FVC		41.9 for children receiving dupilumab; 23.7 in the placebo group

The “×” symbol indicates that the absence of the domain was explicitly required as part of the remission definition for the specified duration. Blank cells indicate that the domain was not included in the study’s remission definition. DeBoer et al^28^ were excluded from this table because no explicit definition of asthma remission was provided. Notably, no study incorporated biologic or inflammatory markers as part of their remission definitions.

*ACQ-5*, Asthma Control Questionnaire 5; *FVC*, forced vital capacity; *ICS*, Inhaled corticosteroids; *JPAC*, Japanese Pediatric Asthma Control questionnaire; *NR*, not reported; *OCS*, oral corticosteroids.

Across studies, the most common remission criteria were the absence of symptoms (6 of 7, excluding DeBoer) and medication discontinuation (5 of 7), although the specific drug classes varied considerably. Cessation of ICS and OCS were each required by 4 studies; however, these were not uniformly paired. For instance, Manuyakorn et al^25^ mandated no ICS use but did not mention OCS, whereas Bacharier et al^22^ required the absence of OCS despite ongoing ICS use. Only 3 studies explicitly required rescue medication discontinuation. Exacerbations (2 of 7) and lung function (3 of 7) were less consistently included, and inflammatory markers were not used. Although remission definitions commonly included symptom control, exacerbations, medication use, and lung function, none of the identified studies incorporated patient-centered outcomes such as health-related quality of life, functional status, or social participation.

### Prevalence of remission

Among 4 studies reporting remission statistics, estimates were highly heterogeneous. In the VOYAGE trial, 41.9% of children with severe asthma on dupilumab achieved clinical remission at 52 weeks versus 23.7% in the placebo group.^22^ Based on a visual estimation from a reported bar graph, Odajima et al^23^ found that approximately 40% of children with severe asthma achieved complete symptom control (Japanese Pediatric Asthma Control questionnaire score 15 of 15) by 24 weeks of treatment with omalizumab.

In a population-based cohort across 4 Canadian provinces, Longo et al^21^ followed 48,687 preschoolers with physician-confirmed moderate-to-severe asthma. Over a median follow-up of 4 years, 46.6% achieved clinical remission. However, our reanalysis focusing specifically on patients with suboptimal asthma control trajectories (characterized as fluctuating, worsening, or out of control) used as proxies for more severe disease showed a lower remission rate of 41.5%. Remission occurred mainly during early school age, peaking at 8-9 years (13.1%), with most occurring between the ages of 6 and 11 years, and only 1.2% before age 6 years. This study used a less-stringent definition of remission based on the absence of health service use, including exacerbations and medications for asthma rather than symptom questionnaires.

In the cohort study by Andersson et al,^27^ 9 of 67 (13.4%) aged between 7 and 19 years with severe asthma achieved remission during a 12-year follow-up period.

### Predictors of remission

Of the 8 included studies, only 3 assessed clinical, hormonal, or inflammatory predictors of asthma remission (Table III).^21^^,^^27^^,^^28^ In Andersson et al,^27^ children with severe asthma who were nonsensitized on skin prick testing at ages 7-8 years had a substantially higher remission probability by age 19 years (32%) compared with only 4% among those with both sensitization and high severity.

**Table III:** Predictors of remission or improved asthma outcomes in children with moderate-to-severe asthma

Study	Population	Outcome domain	Predictor	Measurement period for predictors	Association	Effect estimate (as reported)
Andersson et al (2013)^27^	Children with severe asthma	Clinical remission at 19 y	Negative SPT to furry animals (vs positive)	At the age of 7-8 y	Favorable	Remission: 32% vs 4% *P* < .001
DeBoer et al (2018)^28^	Adolescents with severe asthma	Symptom control (mean ACQ-6)	Males: advanced pubertal maturation, pubic hair (Tanner 3-5 vs 1-2)	At study enrollment	Favorable	0.9 vs 1.5, *P* < .05
			Females: advanced pubertal maturation, pubic hair (Tanner 3-5 vs 1-2)		Unfavorable	1.4 vs 1.1 *P* < .05
			Females: advanced pubertal maturation, breast (Tanner 3-5 vs 1-2)		Unfavorable	1.3 vs 1.1 *P* < .05
Longo et al (2021)^21^	Population-based cohort of preschoolers with asthma	Remission, as defined by no health visits or medication use for asthma over the course of 2 consecutive years	Fluctuating control trajectory vs controlled throughout	All trajectories were ascertained in the 2 y following preschool diagnosis.	Unfavorable	HR 0.76 (95% CI, 0.72-0.82)
			Worsening control trajectory versus controlled throughout		Unfavorable	HR 0.61 (95% CI, 0.56-0.66)
			Out-of-control trajectory vs controlled throughout		Unfavorable	HR 0.47 (95% CI, 0.42-0.51)
			Male sex vs female	At birth	Unfavorable	HR 0.92 (95% CI, 0.90-0.95)
			Age group (y)∗ 1 vs 0 2 vs 0 3 vs 0 4 vs 0	At diagnosis	Unfavorable	HR 0.88 (95% CI, 0.85-0.91) HR 0.79 (95% CI, 0.76-0.82) HR 0.76 (95% CI, 0.73-0.78) HR 0.79 (95% CI, 0.72-0.87)
			Atopy vs none∗	In the 2 y following diagnosis	Unfavorable	HR 0.85 (95% CI, 0.83-0.87)
			≥1 Hospitalization vs none	At diagnosis	Unfavorable	HR 0.89 (95% CI, 0.86-0.92)
			Season∗ Spring vs winter Summer vs winter Fall vs winter	Of diagnosis	Unfavorable	HR 0.96 (95% CI, 0.92-0.99) HR 0.93 (95% CI, 0.89-0.96) HR 0.91 (95% CI, 0.88–0.94)

Effect estimates are reported exactly as presented in the original studies; no statistical pooling or reanalysis was performed. HRs summarized across site-specific estimates reported in the Longo et al^21^ study using a random-effects model.

*ACQ-6*, Asthma Control Questionnaire 6; *CI*, confidence interval; *HR*, hazard ratio; *SPT*, skin prick test.

∗HRs summarized across site-specific estimates reported in the Longo et al study using a random effects model.

In the population-based cohort study by Longo et al,^21^ several characteristics reduced remission likelihood: male sex (hazard ratio [HR], 0.92 [95% CI, 0.90-0.95]), older age at asthma diagnosis (eg, HR, 0.79 [95% CI 0.72-0.87] at 4 years), and atopy (HR, 0.85 [95% CI, 0.83-0.87]). Preschoolers diagnosed with asthma in hospital had a decreased probability of remission (HR, 0.89 [95% CI 0.86-0.92]) during childhood. Season of preschool diagnosis influenced outcomes, with diagnoses in summer (HR, 0.93 [95% CI, 0.89-0.96]) or fall (HR, 0.91 [95% CI, 0.88-0.94]) compared with winter, decreasing the likelihood of achieving remission. Finally, longitudinal control trajectories following diagnosis strongly shaped prognosis. Compared with children whose asthma remained controlled throughout the 2 years after diagnosis, those with suboptimal control trajectories had significantly reduced remission rates. Specifically, likelihood was lower for children with fluctuating (HR, 0.68 [95% CI, 0.60-0.76]), worsening (HR, 0.68 [95% CI, 0.59-0.77]), and persistently out-of-control asthma (HR, 0.49 [95% CI, 0.43-0.55]).

Hormonal predictors were investigated by DeBoer et al,^28^ a prospective cohort of adolescents with moderate-to-severe asthma. Among males in the severe subgroup, advancing pubertal maturation (Tanner stages 3-5 vs 1-2) significantly improved symptom control, reflected by lower ACQ-6 scores (0.9 vs 1.5; *P* < .05). According to recent definitions,^32^ this mean ACQ-6 score of 0.9 in postpubertal males meets the ≤1.5 threshold for clinical remission. In females, however, symptom control worsened slightly with pubertal progression. Advanced maturation increased ACQ-6 scores from 1.1 to 1.4 when defined by pubic hair development (*P* < .05) and from 1.1 to 1.3 when defined by breast development (*P* < .05). Although these absolute scores remain below the 1.5 threshold and technically classify as clinical remission, the upward trajectory could suggest a trend toward uncontrolled asthma for female patients as puberty advances. Regarding lung function, unlike the ACQ-6 symptom scores, the data were not stratified by severe versus nonsevere asthma for the overall cohort.

### Long-term outcomes of remission

Three of the 8 included studies evaluated long-term outcomes after achieving clinical remission in moderate-to-severe asthma. Longo et al^21^ showed that among children who achieved remission, 15.6% subsequently experienced a recurrence, corresponding to 2.4 events per 100 person-years, highlighting that remission is not always sustained. In the cross-sectional study from Lithuania, Pumputiene et al^24^ reported that signs of T cell and eosinophil activation, specifically, elevated serum levels of IL-2 and soluble IL-2 receptor measured via ELISA, alongside eosinophil cationic protein measured via fluoroimmunoassay, were still detectable in children aged between 7 and 10 years who had entered clinical remission, particularly among those with a history of more severe disease. This suggests that underlying immune activation may continue despite symptom resolution, potentially contributing to long-term airway remodeling. Similarly, Manuyakorn et al^25^ found that serum levels of TGF-β1, a profibrotic cytokine indicative of airway remodeling and structural thickening, remained elevated in children with atopic asthma who were asymptomatic at the time of evaluation. These findings suggest that even in clinical remission, underlying pathological processes such as fibrosis may persist.

## Discussion

This scoping review synthesized evidence on remission in pediatric moderate-to-severe asthma. Despite an extensive search, only 8 studies met inclusion criteria, underscoring how rarely remission is investigated in these subgroups. Many screened studies were excluded from grouping transient or mild asthma with moderate-to-severe asthma, which would have diluted findings relevant to this specific population. This limited evidence highlights the need for focused research on remission as a therapeutic goal, where achieving and sustaining it may be more challenging.^33^

Remission definitions were rarely as rigorous as adult multidomain criteria and were heterogeneous, limiting comparability and reducing clinical relevance. Our findings align with recent data showing that pediatric remission definitions are primarily clinical, focusing on symptom absence and medication discontinuation.^19^ Although straightforward to apply, these criteria may overlook key biological or lifestyle subdomains, such as activity limitations, which are important to clinicians and patients.^34^

An important finding of this review was the absence of quality-of-life measures, functional status, and social participation from all identified remission definitions. This may represent a disconnect between currently proposed remission criteria and outcomes that are meaningful to children and their families. Previous studies have shown that parents, caregivers, and young people view quality of life, participation in normal daily activities, and overall wellbeing as important indicators of successful asthma management.^35^^,^^36^ Consequently, future consensus definitions of remission in pediatric asthma should consider incorporating patient-centered outcomes alongside traditional clinical measures such as symptoms, exacerbations, medication use, and lung function.

Our review also highlights that remission in moderate-to-severe asthma is often evaluated in the context of ongoing treatment; we therefore could not distinguish treatment associated from spontaneous remission based on available evidence. Although Anastasiou et al^19^ conceptualized remission as spontaneous symptom resolution across all severities, our findings suggest a different reality for children with severe disease. In this subpopulation, remission is frequently achieved through ongoing therapy, particularly biologics. This shift is critical, as prior studies focused on off-treatment remission in milder asthma.^37^ With biologic therapies central to managing pediatric moderate-to-severe asthma, on-treatment remission becomes a more relevant clinical target for sustainable disease control. Supporting this, the highest remission rates occurred in the biologic trials of Bacharier et al^22^ and Odajima et al.^23^ Rather than natural disease resolution, remission may represent deep, sustained control facilitated by biotherapies. Consequently, focused research on biologics in pediatric moderate-to-severe asthma is critically needed. Although biologic-associated remission is well studied in adults,^38^ only 2 pediatric studies have evaluated this outcome.

Measures such as lung function and biomarkers were inconsistently included or omitted from remission definitions. Clinical remission does not necessarily imply pathophysiologic resolution, and relying solely on symptomatic control may mask persisting disease activity such as abnormal lung function or airway inflammation. This is concerning in asymptomatic children with ongoing inflammation. However, assessing physiologic remission in young children remains challenging because spirometry is difficult to conduct under age 5 years, and airway hyperresponsiveness and inflammation tests are rarely used due to accessibility, technical, and feasibility issues.^1^^,^^39^ The predominance of simple clinical criteria highlights the need for definitions applicable to everyday pediatric decision making.

Our review also highlights a notable absence of studies assessing biological or inflammatory markers as predictors of remission. Among studies evaluating the probability of achieving remission, several clinical patterns emerged. Remission occurred more often in younger children, whereas in adolescence, advanced pubertal maturation was associated with improved symptom control in males but worsening control in females. In contrast, higher initial disease severity, atopic comorbidities, persistent indicators of inadequate control in preschool, and prior hospitalizations were associated with a lower likelihood of remission during childhood.

Although some included studies focused on clinical predictors of remission, none evaluated inflammatory biomarkers. This is noteworthy because type 2 biomarkers such as blood eosinophils, fractional exhaled nitric oxide, and IgE predict future exacerbation risk and morbidity in children, especially in multimarker models.^40^^,^^41^ In severe asthma and biologic-treated populations, blood and sputum type 2 inflammatory signatures help identify “super-responders” and remission after anti–IL-5 therapy, underscoring the link between baseline inflammatory phenotypes and achieving remission.^42^, ^43^, ^44^ This lack of inflammatory predictors represents a critical gap, limiting early precision strategies.^44^, ^45^, ^46^, ^47^

Stratified analyses by age, pubertal stage, and sex were limited, despite their critical role in understanding remission heterogeneity. Remission trajectories differ significantly between sexes due to nonlinear developmental and hormonal changes, including changes in airway anatomy during puberty.^48^, ^49^, ^50^ For instance, boys experience higher remission rates postpuberty, and factors driving remission, such as early FEV_1_/forced vital capacity improvements in females versus reduced eosinophilia in males, are highly sex specific.^51^ Therefore, analyses that pool results across sexes or broad age ranges risk obscuring these distinct remission patterns.

Key strengths of this review include a rigorous methodology, with dual-reviewer screening, standardized extraction, and alignment with a structured scoping framework ensuring reproducibility. A comprehensive multidatabase search further strengthens our findings.

However, this review also had several limitations. At the review level, the small number of eligible studies and substantial heterogeneity in remission definitions, outcomes, and designs limited comparability and subgroup/sensitivity analyses by severity, age group, and sex. Although this likely reflects the relative immaturity of the field and the recent emergence of remission as a clinical and research concept in pediatric moderate-to-severe asthma, search-related limitations should also be considered. Terminology surrounding remission is not yet standardized, and relevant studies may have described similar concepts using alternative terms such as sustained control, disease inactivity, symptom resolution, or long-term disease control. Although a comprehensive search strategy was developed with an experienced librarian and implemented across multiple databases, some studies may not have been identified because they did not explicitly contain the search terms that were agreed on. Consequently, the available literature may be broader than that represented by the studies included in this review. Next, the distinction between treatment associated and spontaneous remission warrants careful consideration. Spontaneous remission has been described primarily in younger children with transient wheeze or milder forms of asthma, where symptom resolution may occur as part of the natural history of the disease. In contrast, the present review focused on children and adolescents with moderate-to-severe persistent asthma, a population in whom remission is generally considered within the context of ongoing or prior treatment. As a result, none of the included studies explicitly differentiated treatment associated from spontaneous remission, limiting our ability to compare these remission states directly. Lastly, as this was a scoping review, we did not conduct a formal assessment of study quality or risk of bias. As a result, our findings should be interpreted as a description of how remission has been defined and studied rather than an evaluation of the strength of the supporting evidence. This limitation may be particularly relevant for smaller studies, which may be more vulnerable to imprecision, selection bias, and limited external validity. Therefore, although we identified substantial heterogeneity in remission definitions, we cannot determine whether certain definitions are supported by higher-quality evidence than others. As the literature on moderate-to-severe pediatric asthma remission matures, future systematic reviews employing formal critical appraisal tools, such as the Mixed Methods Appraisal Tool, are warranted to formally evaluate the methodological quality and risk of bias in this domain.

Although we did not formally assess the quality of included studies, many studies included small sample sizes (n = 38-83),^23^, ^24^, ^25^ whereas cross-sectional designs^24^^,^^25^^,^^28^ can only estimate prevalence but cannot examine the temporal sequence of events. Furthermore, in the preschool cohort study conducted by Longo et al,^21^ eligibility criteria requiring prolonged disease activity to avoid including children with transient viral wheeze may have inadvertently excluded those who achieved early remission. Reliance on administrative data precluded access to symptom scores, lung function, and inflammatory biomarkers, limiting the clinical granularity of the remission definition. In Covar et al,^26^ lacking a moderate asthma subgroup is a key limitation. Although the cohort included both mild and moderate persistent asthma, predictors were analyzed across the full sample, potentially obscuring severity-specific insights. Andersson et al^27^ used a nonvalidated severity score based on symptoms and medication use, without accounting for oral corticosteroid exposure. We interpreted a score of ≥2 as indicative of moderate-to-severe asthma. This contrasts with the standard Global Initiative for Asthma definition, which retrospectively classifies severity based on the treatment intensity (Steps 3-5) required for symptom control.^1^ Finally, in the VOYAGE trial,^22^ remission was assessed post hoc rather than as a prespecified outcome, limiting causal inference and increasing the potential for bias.

Importantly, current definitions of remission rely almost exclusively on traditional clinical metrics, largely overlooking the perspectives of children and their families. A review of 4 qualitative research articles highlights recurring patient-centered themes: a desire for normalcy and peer conformity,^52^ a focus on day-to-day symptom relief and a responsive care journey, a preference for individualized goals over one-size-fits-all biomedical targets, and concerns regarding the daily burden and side effects of long-term medication.^53^ Currently, these perspectives are rarely considered when establishing therapy and remission goals. This critical gap must be addressed in future studies by integrating patient-reported outcome measures into standard remission criteria.

Remission in children with moderate-to-severe asthma is an emerging but inconsistently defined outcome. Current evidence supports that clinical remission is achievable in this population. However, a paradigm shift is required. Clinicians should consider on-treatment goals, particularly in the context of biologic therapies, rather than focusing on the spontaneous absence of symptoms. In moderate-to-severe disease, durable control is evaluated with ongoing treatment. Important knowledge gaps remain regarding the predictors of remission and the long-term stability of its subdomains. Future research should prioritize multidimensional, standardized, and validated remission criteria as well as report outcomes separately for children and adolescents. Incorporating biomarkers and evaluating long-term sustainability will be essential to advancing precision-based approaches that position remission as a therapeutic target for children with moderate-to-severe asthma.

## Disclosure statement

C. Longo has received financial support from the 10.13039/501100024252Faculty of Pharmacy at the Université de Montréal, Fonds de Recherche Québec-Santé (FRQS), 10.13039/501100019217Institut de valorisation des données (IVADO), the 10.13039/501100000024Canadian Institutes of Health Research (CIHR), the Canadian Allergy, Asthma, and Immunology Foundation (CAAIF), the Centre Hospitalier Universitaire Sainte-Justine Foundation, and the Lung Health Foundation. S. M. Tse has received unrestricted research funds from 10.13039/100004339Sanofi and 10.13039/100004325AstraZeneca, the Canadian Institute of Health Research, the Quebec Respiratory Health Research Network, and the Institut national de la santé publique du Québec; salary support from the 10.13039/501100000156Fonds de Recherche du Québec - Sante; and honorarium as an invited speaker from the Association des pédiatres du Québec and the Réseau québécois d’éducation en santé respiratoire.

Disclosure of potential conflict of interest: The authors declare that they have no relevant conflicts of interest.

## References

[1] Global Initiative for Asthma, Committee GS, Global strategy for asthma management and prevention, 2024, Global Initiative for Asthma; Fontana, WI, Available from https://ginasthma.org/wp-content/uploads/2024/05/GINA-2024-Strategy-Report-24_05_22_WMS.pdf. Accessed September 23, 2026.

[2] Rutter C, Silverwood R, Fernández VP, Pearce N, Strachan D, Mortimer K, et al. Global Burden of Disease due to asthma, In: *The global asthma report 2022 [Internet]*, 2022, Global Asthma Network; Auckland (NZ), Available from: https://globalasthmareport.org/resources/Global_Asthma_Report_2022.pdf. Accessed September 23, 2026.

[3] FitzGerald J.M., Lemière C., Lougheed M.D., Ducharme F.M., Dell S.D., Ramsey C. (2017). Recognition and management of severe asthma: a Canadian Thoracic Society position statement. Can J Respir Crit Care Sleep Med.

[4] Pate C.A., Zahran H.S. (2024). The status of asthma in the United States. Prev Chronic Dis.

[5] Calhoun W.J., Haselkorn T., Miller D.P., Omachi T.A. (2015). Asthma exacerbations and lung function in patients with severe or difficult-to-treat asthma. J Allergy Clin Immunol.

[6] Rennard S.I., Farmer S.G. (2004). Exacerbations and progression of disease in asthma and chronic obstructive pulmonary disease. Proc Am Thorac Soc.

[7] Lange P., Celli B., Agusti A., Boje Jensen G., Divo M., Faner R. (2015). Lung-function trajectories leading to chronic obstructive pulmonary disease. N Engl J Med.

[8] Papi A., Blasi F., Canonica G.W., Morandi L., Richeldi L., Rossi A. (2020). Treatment strategies for asthma: reshaping the concept of asthma management. Allergy Asthma Clin Immunol.

[9] National Center for Biotechnology Information, Airway remodeling, 2006, National Library of Medicine (US), National Institutes of Health; Bethesda, MD, Available from: https://www.ncbi.nlm.nih.gov/books/NBK7223/. Accessed September 23, 2026.

[10] Smolen J.S., Aletaha D., Bijlsma J.W.J., Breedveld F.C., Boumpas D., Burmester G. (2010). Treating rheumatoid arthritis to target: recommendations of an international task force. Ann Rheum Dis.

[11] Shackleford A., Heaney L.G., Redmond C., McDowell P.J., Busby J. (2025). Clinical remission attainment, definitions, and correlates among patients with severe asthma treated with biologics: a systematic review and meta-analysis. Lancet Respir Med.

[12] Taussig L.M., Wright A.L., Holberg C.J., Halonen M., Morgan W.J., Martinez F.D. (2003). Tucson children's respiratory study: 1980 to present. J Allergy Clin Immunol.

[13] Covar R.A., Fuhlbrigge A.L., Williams P., Kelly H.W. (2012). The Childhood Asthma Management Program (CAMP): contributions to the understanding of therapy and the natural history of childhood asthma. Curr Respir Care Rep.

[14] Thomas D., McDonald V.M., Pavord I.D., Gibson P.G. (2022). Asthma remission: what is it and how can it be achieved?. Eur Respir J.

[15] Lommatzsch M., Virchow J.C. (2025). Asthma remission: a call for a globally standardised definition. Lancet Respir Med.

[16] Lugogo N.L., Mohan A., Akuthota P., Couillard S., Rhoads S., Wechsler M.E. (2023). Are we ready for asthma remission as a clinical outcome?. Chest.

[17] Canonica G.W., Blasi F., Carpagnano G.E., Guida G., Heffler E., Paggiaro P. (2023). Severe Asthma Network Italy definition of clinical remission in severe asthma: a Delphi consensus. J Allergy Clin Immunol.

[18] Miculinic A., Mekie Kobal I., Kusan T., Turkalj M., Plave D. (2024). Current challenges in pediatric asthma. Children (Basel).

[19] Anastasiou E., Miligkos M., Adachi Y., Ansotegui I.J., Badellino H.A., Bekiaris S. (2026). The definition of asthma remission in children: a scoping review. World Allergy Organ J.

[20] Tricco A., Lillie E., Zarin W., O'Brien K.K., Colquhoun H., Levac D. (2018). PRISMA extension for scoping reviews (PRISMA-ScR): checklist and explanation. Ann Intern Med.

[21] Longo C., Blais L., Brownell M., Quail J.M., Sadatsafavi M., Forget A. (2021). Association between asthma control trajectories in preschoolers and disease remission. Eur Respir J.

[22] Bacharier L., Guilbert T.W., Maspero J., Gappa M., Dell S., Altincatal A. (2024). Late Breaking Abstract: clinical remission with dupilumab in children with uncontrolled, moderate-to-severe, type 2 asthma. Eur Respir J.

[23] Odajima H., Ebisawa M., Nagakura T., Fujisawa T., Akasawa A., Ito K. (2017). Long-term safety, efficacy, pharmacokinetics, and pharmacodynamics of omalizumab in children with severe uncontrolled asthma. Allergol Int.

[24] Pumputiene I., Emuzyte R., Dubakiene R., Firantiene R., Tamosiunas V. (2006). T cell and eosinophil activation in mild and moderate atopic and nonatopic children’s asthma in remission. Allergy.

[25] Manuyakorn W., Kamchaisatian W., Atamasirikul K., Sasisakulporn C., Direkwattanachai C., Benjaponpitak S. (2008). Serum TGF-β1 in atopic asthma. Asian Pac J Allergy Immunol.

[26] Covar R., Strunk R., Zeiger R., Wilson L.A., Liu A.H., Weiss S. (2010). Predictors of remitting, periodic, and persistent childhood asthma. J Allergy Clin Immunol.

[27] Andersson M., Hedman L., Bjerg A., Forsberg B., Lundbäck B., Rönmark E. (2013). Remission and persistence of asthma followed from 7 to 19 years of age. Pediatrics.

[28] DeBoer M., Phillips B., Mauger D., Zein J., Erzurum S., Fitzpatrick A.M. (2018). Effects of endogenous sex hormones on lung function and symptom control in adolescents with asthma. BMC Pulm Med.

[29] Kondo N., Nishimuta T., Nishima S., Morikawa A., Aihara Y., Akasaka T. (2010). Japanese pediatric guidelines for the treatment and management of bronchial asthma 2008. Pediatr Int.

[30] Zeiger R., Dawsonb C., Weiss S. (1999). Relationships between duration of asthma and asthma severity among children in the Childhood Asthma Management Program (CAMP). J Allergy Clin Immunol.

[31] Matsunaga M., Sato Y., Nagao M., Ikeda M., Motomura C., Kameda M. (2024). Development and validation of a new asthma questionnaire to help achieve a high level of control in school-age children and adolescents. Allergol Int.

[32] Thawanaphong S., Nolasco S., Nair P. (2025). Achieving remission in severe asthma. Chin Med J Pulm Crit Care Med.

[33] Ross K.R., Gupta R., DeBoer M.D., Zein J., Phillips B.R., Mauger D.T. (2020). Severe asthma during childhood and adolescence: a longitudinal study. J Allergy Clin Immunol.

[34] Bradley H., Trennery C., Jones A.M., Lydon A., White F., Williams-Hall R. (2013). Assessing asthma symptoms in children: qualitative research supporting the development of the Pediatric Asthma Diary-Child (PAD-C) and Pediatric Asthma Diary-Observer (PAD-O). J Patient Rep Outcomes.

[35] Sinha I.P., Gallagher R., Williamson P.R., Smyth R.L. (2012). Development of a core outcome set for clinical trials in childhood asthma: a survey of clinicians, parents, and young people. Trials.

[36] Kelada L., Molloy C.J., Hibbert P., Wiles L.K., Gardner C., Klineberg E. (2021). Child and caregiver experiences and perceptions of asthma self-management. NPJ Prim Care Respir Med.

[37] Menzies-Gow A., Bafadhel M., Busse W.W., Casale T.B., Kocks J.W.H., Pavord I.D. (2020). An expert consensus framework for asthma remission as a treatment goal. J Allergy Clin Immunol.

[38] Busse W.W. (2024). The role of biologics in inducing remission in asthma. Ann Allergy Asthma Immunol.

[39] Global Initiative for Asthma (2025).

[40] Meulmeester F., Mailhot-Larouche S., Celis-Preciado C. (2025). Inflammatory and clinical risk factors for asthma attacks (ORACLE2): a patient-level meta-analysis of control groups of 22 randomised trials. Lancet Respir Med.

[41] Konradsen J., Skantz E., Nordlund B., Lidegran M., James A., Ono J. (2015). Predicting asthma morbidity in children using the proposed markers of Th2-type inflammation. Pediatr Allergy Immunol.

[42] Moermans C., Brion C., Bock G., Graff S., Gerday S., Nekoee H. (2023). Sputum type 2 markers could predict remission in severe asthma treated with anti-interleukin-5. Chest.

[43] Principe S., Porsbjerg C., Bolm Ditlev S., Kjærsgaard Klein D., Golebski K., Dyhre-Petersen N. (2021). Treating severe asthma: targeting the IL-5 pathway. Clin Exp Allergy.

[44] Mailhot-Larouche S., Celis-Preciado C., Heaney L., Couillard S. (2024). Identifying super-responders: a review of the road to asthma remission. Ann Allergy Asthma Immunol.

[45] Licari A., Manti S., Castagnoli R., Leonardi S., Marseglia G. (2020). Measuring inflammation in paediatric severe asthma: biomarkers in clinical practice. Breathe.

[46] Uwaezuoke S., Ayuk A., Eze J. (2018). Severe bronchial asthma in children: a review of novel biomarkers used as predictors of the disease. J Asthma Allergy.

[47] Scotney E., Burchett S., Goddard T., Saglani S. (2021). Pediatric problematic severe asthma: recent advances in management. Pediatr Allergy Immunol.

[48] Vink N., Postma D., Schouten J., Rosmalen J., Boezen H. (2010). Gender differences in asthma development and remission during transition through puberty: the TRacking Adolescents' Individual Lives Survey (TRAILS) study. J Allergy Clin Immunol.

[49] Trivedi M., Denton E. (2019). Asthma in children and adults—what are the differences and what can they tell us about asthma?. Front Pediatr.

[50] Shah R., Newcomb D. (2018). Sex bias in asthma prevalence and pathogenesis. Front Immunol.

[51] Wang H., Lin X., Hao C., Zhang C., Sun B., Zheng J. (2006). A double-blind, placebo-controlled study of house dust mite immunotherapy in Chinese asthmatic patients. Allergy.

[52] Callery P., Milnes L., Verduyn C., Couriel J. (2003). Qualitative study of young people's and parents' beliefs about childhood asthma. Br J Gen Pract.

[53] Kassa E., Kebede R.A., Habte B.M. (2022). Perceptions towards childhood asthma and barriers to its management among patients, caregivers and healthcare providers: a qualitative study from Ethiopia. BMC Pulm Med.

